# Neuropharmacological Modulation of N-methyl-D-aspartate, Noradrenaline and Endocannabinoid Receptors in Fear Extinction Learning: Synaptic Transmission and Plasticity

**DOI:** 10.3390/ijms24065926

**Published:** 2023-03-21

**Authors:** Simone Battaglia, Chiara Di Fazio, Carmelo M. Vicario, Alessio Avenanti

**Affiliations:** 1 Center for Studies and Research in Cognitive Neuroscience, Department of Psychology “Renzo Canestrari”, Cesena Campus, Alma Mater Studiorum Università di Bologna, 47521 Cesena, Italy; 2 Department of Psychology, University of Turin, 10124 Turin, Italy; 3 Dipartimento di Scienze Cognitive, Psicologiche, Pedagogiche e Degli Studi Culturali, Università Degli Studi di Messina, 98122 Messina, Italy; 4 Neuropsicology and Cognitive Neuroscience Research Center (CINPSI Neurocog), Universidad Católica del Maule, Talca 3460000, Chile

**Keywords:** neuropharmacology, glutamatergic receptor N-methyl-D-aspartate (NMDA), noradrenaline (NA), enzyme fatty acid amide hydrolase (FAAH), synaptic receptors, brain plasticity, fear learning, fear extinction learning, threat learning

## Abstract

Learning to recognize and respond to potential threats is crucial for survival. Pavlovian threat conditioning represents a key paradigm for investigating the neurobiological mechanisms of fear learning. In this review, we address the role of specific neuropharmacological adjuvants that act on neurochemical synaptic transmission, as well as on brain plasticity processes implicated in fear memory. We focus on novel neuropharmacological manipulations targeting glutamatergic, noradrenergic, and endocannabinoid systems, and address how the modulation of these neurobiological systems affects fear extinction learning in humans. We show that the administration of N-methyl-D-aspartate (NMDA) agonists and modulation of the endocannabinoid system by fatty acid amide hydrolase (FAAH) inhibition can boost extinction learning through the stabilization and regulation of the receptor concentration. On the other hand, elevated noradrenaline levels dynamically modulate fear learning, hindering long-term extinction processes. These pharmacological interventions could provide novel targeted treatments and prevention strategies for fear-based and anxiety-related disorders.

## 1. Introduction

Fear learning is a cross-species ability crucial for survival, as it can promote the avoidance of potentially dangerous situations [1,2,3]. This form of learning allows individuals to predict a dangerous outcome using contextual information or environmental cues and select the safest and most appropriate reaction, expressing species-specific fear responses [4,5]. The neurobiological mechanisms of fear learning have been extensively investigated in several species using Pavlovian conditioning procedures [3,4,5]. Fear conditioning occurs when a neutral stimulus (NS) is paired with an aversive/threatening stimulus—the so-called unconditioned stimulus (US). Following repeated presentations of the NS followed by the US, the NS acquires the capacity to elicit a conditioned fear response, becoming a conditioned stimulus (CS+), and the association between the CS and US is automatically strengthened. In humans, the regulation of emotional responses to potential threats is critical for mental health, and deficits in emotion regulation may lead to trauma-related diseases, such as anxiety and mood disorders [5,6,7,8,9,10,11,12,13,14].

Previously learned fear memories become labile when an event is recalled and may go through two different processes: reconsolidation or fear extinction [5,15,16]. Reconsolidation is the process through which previously consolidated fear memory traces become more sensitive to alterations when reactivated [6,17,18]. This phenomenon implies that a short CS-alone trial leads to the reactivation of a fear memory. This reactivation initiates a temporary destabilization of the memory, which becomes labile. In these states, the memory of the original CS–US association can be more easily modified, strengthened, attenuated, or erased, before reconsolidating and becoming stable again [19]. Indeed, neurobiological interventions during or immediately after the memory reactivation period may affect reconsolidated memories [18]. 

Fear extinction occurs when a consolidated memory is recalled, but no longer reflects significant aversive information. During extinction training, repeated presentations of the CS in the absence of the US result in a reduction in the conditioned fear response [1,20,21]. It is widely accepted that extinction does not reflect memory erasure or unlearning, but rather a new learning process of a CS–no-event contingency [22,23,24], which competes with the original CS–US association in determining behavior during a retention test. This view of extinction has been supported by the demonstration of recovered fear responses after extinction, or by the spontaneous recovery of extinguished fear memories [25]. In this vein, a new fear extinction memory inhibits the CS–US association by updating the original CS–US fear memory, or suppressing the original memory trace, instead of deleting it during the extinction process (i.e., re-learning) [8,9,26,27,28]. 

In humans, it has been proposed that fostering the extinction of fear memories can be an effective therapeutic strategy to control emotional responses [5,13,29]. In a neuropharmacological context, the facilitation of fear extinction is achieved using relevant pharmacological adjuvants targeting N-methyl-D-aspartate (NMDA), noradrenaline, and endocannabinoid receptors that act on neurochemical systems implicated in fear memory processes [29,30]. Such interventions have been experimentally and clinically tested with the ultimate goal of facilitating re-learning, thus reducing the expression of behavioral and physiological fear responses [31,32,33,34,35,36,37]. Accordingly, these pharmacological enhancers promote the natural response of the organism by facilitating the release of neurotransmitters within a specific regulatory system implicated in fear extinction [33,38,39]. 

It has been widely reported that these neuropharmacological adjuvants act on neurochemical synaptic transmission and brain plasticity processes implicated specifically in fear extinction learning. Because of their acknowledged role in synaptic transmission and plasticity, the ionotropic *glutamate receptors* have been a major focus of research in human neuropharmacological studies of memory recall and extinction [40]. Indeed, glutamate-mediated neurons are widely distributed in the amygdala, hippocampus, and other brain regions critically involved in associative and fear learning [41,42,43,44]. Ionotropic glutamate receptors are classified into three sub-families based on their affinity for synthetic agonists: α-amino-3-hydroxy-5-methyl-4-isoxazole propionate (AMPA), NMDA, and kainate [40,45,46]. AMPA receptors are known to mediate fast synaptic responses [47] and NMDA receptors to mediate slow synaptic responses at most excitatory synapses in the brain [48]. Kainate receptors are formed from a separate set of genes (GluR5–7, KA-1, and KA-2) and are known to be implicated in epileptogenesis and cell death [49]. Research indicates that AMPA receptors contribute to conditioned fear in the amygdala, as infusions of AMPA receptor antagonists into this region interfere with the expression of conditioned fear [50,51]. Furthermore, hippocampal AMPA and kainate receptors have been implicated in regulating spatial memory, fear acquisition, and other forms of learning and memory [52,53]. Changes to the expression of AMPA and kainate subunits in the hippocampus, prefrontal cortex, and amygdala may, therefore, partially explain deficits in contextual fear learning [53]. 

NMDA receptor-dependent neuronal plasticity is a key component of the emotional learning process [54,55]. Human studies involving the administration of NMDA receptor antagonists, particularly GluN2B knockout, support the involvement of this subfamily of receptors in memory consolidation and in the extinction of fear memory [56], showing a dose-dependent impairment of these processes. While NMDA receptor antagonists impair extinction, the receptor partial agonist D-cycloserine (DCS) facilitates this process. DCS acts at the glycine modulatory site on the NR1-NMDA receptor subunit to increase calcium influx without causing neurotoxicity-induced damage. Therefore, it is considered a promising cognitive enhancer in humans, as it reverses the influence of fear extinction learning [57,58,59], with a positive therapeutic effect on most anxiety disorders, such as simple phobia [60], social phobia [61,62], panic disorder [63], and obsessive-compulsive disorder [64].

In addition to glutamate receptors, the *noradrenergic receptors* are one of the most intensively studied classes of metabotropic receptors in behavioral responses to stressful events, especially for their role in fear memory. Noradrenergic receptors are classified as either alpha or beta receptors. Those two classes are further subdivided into alpha-1, alpha-2, beta-1, beta-2, and beta-3. Alpha-1 and alpha-2 receptors each have three subtypes. More specifically, α-adrenoceptors are presynaptic autoreceptors involved in the regulation of noradrenaline (norepinephrine) release; β-receptors, along with beta-2, alpha-1, and alpha-2 receptors, are adrenergic receptors primarily responsible for signaling in the sympathetic nervous system and have long been associated with fear disorders, as well as learning and memory [65,66]. In the central nervous system, α- and β-receptors can also be found at a postsynaptic level, with evidence suggesting that *α*-adrenoceptors are affected by stress [67]. The two α-receptor subtypes, α1 and α2, have been implicated in fear learning. Typically, the inhibition of the α1 receptor selectively reduces fear learning, improving the extinction of fear memory. The antagonism of the α1 receptor leads to poor performance in fear learning tasks. For instance, prazosin, an α1 antagonist, has been shown to reduce fear responses in both olfactory fear paradigms and olfactory recall tasks [68]. The α1 and α2 receptors are implicated in different fear learning mechanisms, as the antagonism of α2 receptors significantly improves memory and cognition in a variety of contexts. Indeed, the antagonist yohimbine, which works by decreasing the inhibitory influence of the α2 receptor on noradrenaline release, promotes fear-conditioned responses.

Furthermore, the *endocannabinoid system* has also been widely studied in fear extinction processes, with endocannabinoids (eCBs) being released in response to specific physiological needs and pharmacologically increased by blocking catabolic degradation [69,70]. The most well-known eCBs are 2-arachidonoyl glycerol (2-AG) and anandamide (AEA), which can be pharmacologically augmented by blocking their reuptake from the extracellular space or by inhibiting endocannabinoid-degrading enzymes to avoid their catabolic degradation. These neurotransmitters are predominantly degraded by the catabolic enzymes fatty acid amide hydrolase (FAAH) and monoacylglycerol lipase, respectively, which produce distinct behavioral effects [71,72]. In particular, evidence shows that the chronic inhibition of monoacylglycerol lipase causes physical dependence, impaired endocannabinoid-mediated synaptic plasticity, and cannabinoid receptor desensitization, while the chronic inhibition of FAAH selectively boosts endogenously recruited anandamide in corticolimbic circuits, promoting extinction recall after successful fear learning and extinction [69,73].

A significant amount of recent research has identified neurotransmitters and synaptic receptors involved in the extinction of fear memory [74,75,76,77]. Namely, due to their established role in synaptic transmission and plasticity, both glutamate and endocannabinoid receptors have been widely investigated in mechanisms underlying the depotentiation of original fear memory. However, there is still debate about the effects of noradrenaline antagonists, as their actions in the presynaptic terminals can promote noradrenaline release [78,79,80]. In particular, β-adrenergic receptors have been reported to modulate fear extinction by enhancing the acquisition rate of the inhibitory extinction memory [81,82]. Still, it is unclear whether increases in noradrenergic arousal could conceptually support the enhanced acquisition of extinction learning or foster its consolidation [31,83,84]. 

Hence, our aim is to report and discuss pertinent mechanisms behind human models of fear extinction, with a focus on studies that have developed pharmacological methods to facilitate fear extinction learning in humans by tracking synaptic transmission and plasticity. This review discusses the advances of the last decade that have been made in this field by presenting the mechanisms of action of pharmacological adjuvants in the modulation of NMDA, noradrenaline, and endocannabinoid receptors. We will firstly present evidence supporting the hypothesis that NMDA antagonists may have an excitatory role in human fear extinction by inhibiting US memory. Then, the modulation of the noradrenaline and endocannabinoid anandamide levels in extinction learning will be reviewed. In light of this, we will discuss the relevance of aversive memory extinction for the study of fear inhibition and for the screening of putative pharmacotherapies for psychiatric clinical settings.

## 2. The Effects of NMDA Agonist D-Cycloserine (DCS) and Valproic Acid (VPA) 

Glutamatergic NMDA receptors have long been reported to be involved in the acquisition [85,86,87], consolidation, and extinction of fear memories [88,89]. Indeed, the NMDA receptor agonist D-cycloserine (DCS) is one of the most well-studied pharmacological adjuvants [90,91]. DCS has been shown to act as a potential cognitive enhancer for the treatment of anxiety disorder, phobias, obsessive-compulsive behavior, and schizophrenia [92,93,94]. The NMDA glutamate receptor’s function can be enhanced by stimulating one of the high-affinity glycine binding sites, a feature of the NMDA–glutamate receptor complex [95]. D-cycloserine (DCS) is a partial agonist of the glycine site and indirectly increases glutamatergic activity in previously “silent” synapses [96]. When the surrounding glycine levels are low, it facilitates NMDA receptor function with up to approximately 60% of the efficacy of glycine, thereby increasing neuroplasticity and interfering with the consolidation of fear memories. Both processes are thought to facilitate fear extinction [97,98]. Although NMDA receptors are crucial for the initial acquisition of fear [21,22,37,41,44,99,100], their role in extinction learning has only been investigated in recent years [62,100,101]. Growing evidence suggests that fear extinction can be enhanced by adjuvant neuropharmacological therapies [33,102], particularly treatment approaches targeting NMDA receptors. Those approaches have been investigated as pharmacotherapies that increase fear extinction, thus enhancing the efficacy of extinction-based psychotherapies [60,101,103]. Additionally, it has been shown that NMDA antagonists enhance extinction through some form of inhibition of the US memory, rather than by disrupting the consolidation of conditioned responses [99]. In particular, it has been reported that DCS administration is involved in the treatment of fear-related disorders, as its modulation can enhance extinction learning in patients with different types of anxiety disorders (i.e., post-traumatic stress disorder, phobias, panic disorders, social anxiety, and obsessive-compulsive disorder) [60,102,104]. Accordingly, animal studies suggest that DCS can facilitate the extinction of fear learning [90,105], reduce the reinstatement of fear memories after the presentation of a single US [31,106,107], and promote the generalization of extinction from one CS to another [108,109]. 

To evaluate the efficacy of DCS for enhancing the effects of fear learning extinction, Kuriyama et al. [110] examined the physiological and pharmacological effects of administering DCS and valproic acid (VPA) on the extinction of fear learning using a re-exposure paradigm. Previous studies have demonstrated that VPA—an anticonvulsant that modulates GABA and glutamate-mediated neurotransmission by acting on multiple mechanisms, including the inhibition of histone deacetylase (HDAC) [111], and increasing the messenger RNA (mRNA) and protein levels of brain-derived neurotrophic factor (BDNF) [112]—increases extracellular dopamine levels in the medial prefrontal cortex and hippocampus [113], a mechanism that is believed to modulate the expression and suppression of learned fear responses. Consequently, to examine the effects of these two drugs on the extinction of fear learning, participants (n = 59) were randomly assigned to four groups based on the pharmacological treatment they received: a DCS group, a VPA group, a combined VPA–DCS group, and a placebo group. The experimental design consisted of three consecutive days. On day 1, during the acquisition session, one CS+ (i.e., a geometric figure) was paired with an aversive stimulus (i.e., mild electrical stimulation; US), while two CS- (i.e., geometric figures) were never presented with the US. On day 2, during a new acquisition session, one previous CS- was paired with the US, along with the other CS- and CS+ that were presented without the US (i.e., extinction). Crucially, on the same day, 90 min before the acquisition session, distinct groups of participants were administered a placebo, 100 mg of DCS, 400 mg of VPA, or a combination of 100 mg of DCS and 400 mg of VPA, to examine the effects of DCS and VPA alone or in combination on extinction learning. Finally, on day 3, there was a test session that included extinction recall and reinstatement phases. During the extinction recall phase, all the CSs were delivered without the US to test the effects of DCS and VPA, while the two CS+ were paired with the US during the reinstatement phase. The skin conductance response (SCR) was used to assess fear responses across groups. The results showed that either a single dose of DCS or VPA, or a combination of DCS and VPA interfered with the acquisition of fear memory on day 2, promoting memory extinction and reducing the reinstatement effect. These results demonstrated that, even though DCS has been shown to enhance fear memory consolidation [62,114] by enhancing subsequent conditioned fear responses, in combination with VPA, it can also promote the extinction of fear learning and prevent the relearning of previously conditioned fear responses. 

In a subsequent study, Kuriyama et al. [115] administered DCS and VPA to determine whether these neuropharmacological treatments mainly interfere with fear acquisition or with the delayed consolidation of fear memory. Participants (n = 87) were randomly assigned to six groups based on the pharmacological treatment received and on their wake–sleep state. The study was conducted on two consecutive days: on day 1, participants were fear conditioned with one of three geometric figures presented as a CS+ and paired with a mild electric shock (US). On the same day, the DCS group received 100 mg of DCS, the VPA group received 400 mg of VPA, and the placebo group received 1000 mg of lactose 1 h before the second acquisition session, which occurred after a 2 h interval. In the second fear acquisition session, a different CS+, randomly selected from the prior CS- stimulus set, was presented with the US, along with other CSs that were not paired with the US. On day 2, after a 12 h interval that included a waking period with or without habitual sleep, the authors assessed the effects of the pharmacological treatment through extinction recall: participants were reminded of all the CSs through nine presentations without the US. Immediately afterward, during the reinstatement phase, the two CS+ were delivered with the US. SCR was used to assess fear responses. The results showed that VPA attenuated fear responses to the CS+ during the second fear-acquisition session and blocked the reinstatement of fear learning. Additionally, VPA reduced fear expressions following habitual sleep, while DCS blocked the effect following a waking period. These data demonstrate that DCS enhanced ‘offline’ extinction learning during the waking state, whereas VPA had a delayed effect on fear learning extinction, rather than an instant regulation effect, probably because fear responses were reduced after the habitual sleep period in the post-reinstatement session.

In a similar attempt, Ebrahimi et al. [116] investigated the effect of DCS augmentation on extinction learning using functional magnetic resonance imaging (fMRI). Participants (n = 37) were randomly assigned to the DCS group and placebo group. The experimental design was divided into three consecutive days: on day 1, the participants were fear-conditioned with two different CSs (i.e., two male faces), of which one was paired with a loud noise (US). On day 2, an oral dose of 50 mg of DCS or placebo was administered once before extinction learning, during which neither of the two CSs were paired with the US. Finally, on day 3, participants underwent the extinction recall phase, in which non-reinforced CS+ cues were presented. Subjective ratings of valence and arousal were collected after the fear acquisition session, and both SCR and fMRI were used as dependent variables to assess fear responses. The results showed no group differences in fear acquisition, as indicated by enhanced arousal ratings, as well as the activation of the insula, dorsal anterior cingulate cortex (dACC), and thalamus in response to the CS+ presentation. However, only the placebo group showed generalization of subjective and psychophysiological conditioned responses to the CS+, suggesting that DCS enhanced extinction learning and prevented fear consolidation. These data were also supported by a decrease In amygdala activity in the DCS group during extinction recall, while the placebo group had greater dACC, insula, and posterior hippocampal activation in response to the CS+. 

**Figure 1:**
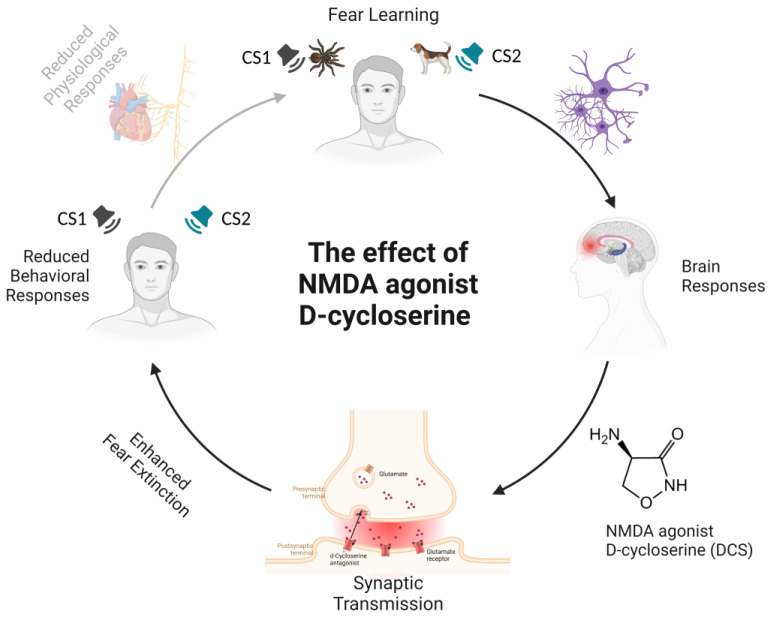
**Schematic representation of the amelioration of N-methyl-D-aspartate (NMDA) receptor activity-related effects by the administration of agonist D-cycloserine (DCS) during fear extinction.** During a fear-learning paradigm, a previously neutral stimulus (the conditioned stimulus) acquires emotional significance through pairing with an aversive stimulus. The aversive stimulus elicits a range of automatic, unconditioned fear responses, such as freezing and increased heart rate or blood pressure. After a few pairings, the presentation of the conditioned stimulus alone is capable of eliciting a conditioned fear response. The neural networks underlying fear learning mainly include the amygdala, prefrontal cortex, and hippocampus. Connections between these cortical and subcortical brain regions regulate the acquisition and extinction of fear. The activation of NMDA receptors—ionotropic channels that allow calcium ions into the cell—is necessary for long-term potentiation processes underlying fear learning. DCS, an NMDA partial antagonist, binds to one of the subunits of the NMDA receptor complex and changes its shape. The result is that glutamate opens up the channel and lets more calcium in, leading to boosted excitation by raising the glutamate levels in the interneurons. The specific effects of DCS in humans include enhanced fear extinction memory retention, expressed as attenuated conditioned responses during extinction recall on subjective (i.e., valence and arousal ratings) and physiological (SCR, BOLD response) levels. Notes. CS1 = Conditioned Stimulus 1; CS2 = Conditioned Stimulus 2; DCS = D-cycloserine; NMDA = N-methyl-D-aspartate.

Taken together, these findings provide crucial evidence that enhancing NMDA receptor function by administering DCS improves fear extinction (see Figure 1), as also demonstrated by recent studies on post-traumatic stress disorder (PTSD) patients [117,118,119].

Indeed, such studies have highlighted the mechanisms of human fear extinction promoted by DCS, considering the dosages and timing of administration, thus confirming the potential beneficial effects of DCS in extinguishing conditioned fear by delaying fear memory consolidation [90]. Moreover, the effects of VPA on the extinction of conditioned fear are similar to those of DCS. VPA, as a competitive inhibitor of histone deacetylase proteins, alters the formation of transcription factors, receptors, and other cellular substrates that have a role in plasticity and learning [120]. VPA administration, therefore, results in dendritic sprouting and increases in synaptic connectivity, which modulate learning and promote fear-extinction mechanisms [121] (see Table 1). 

## 3. Effects of Noradrenaline (NA) Modulation 

It is widely recognized that negative and stressful experiences trigger the release of many hormones, neurotransmitters, and peptides [122,123,124]. Among these, noradrenaline (NA) controls neural excitability during the consolidation of fear learning, and plays a specific role in the retrieval of contextual fear memory and reconsolidation processes [124,125,126]. In light of this, recent studies have investigated the possibility of interfering with fear memories using pharmacological manipulations of NA with antagonists and agonists [18,127]. Although NA antagonists can either disrupt or enhance fear memory consolidation in animals [127,128], evidence of the role of NA in fear consolidation and extinction in humans is still unclear and controversial. Critically, according to Kindt et al. [18], blocking ß-adrenergic receptors using propranolol disrupted the reconsolidation of conditioned fear; yet, in later studies, the same authors reported that enhancing NA transmission using the a2-adrenergic antagonist yohimbine could strengthen fear memories, as indicated by delayed extinction learning and facilitated the return of fear [129,130]. 

The effect of NA stimulation on extinction learning and reinstatement of fear responses was extensively investigated in a study by Soeter and Kindt [131]. The experimental design was divided over three consecutive days. On day 1, participants (n = 30) were randomly assigned to the test group and the placebo group. They were fear-conditioned with two different CSs (i.e., images of a spider and a gun) paired with mild electrical stimulation (US). The same day, fear memory consolidation was manipulated by administering 20 mg of yohimbine, an adrenergic receptor antagonist that is meant to stimulate noradrenergic activity by blocking α2 autoreceptors. On day 2, the participants received 40 mg of β-adrenergic receptor antagonist propranolol before memory reactivation, in which a single unreinforced CS+ was presented. Finally, on day 3, the participants were exposed to the CSs without the US during extinction learning, and after the extinction session, an unsignaled shock was presented to reinstate the expression of fear memory. SCR and US ratings were collected to assess fear and extinction learning. The results showed that extinction learning was attenuated after enhancing noradrenergic activity with the antagonist yohimbine, which counteracted the inhibitory action mediated by α2 autoreceptors enhancing the consolidation of fear memory. Additionally, the administration of α2-adrenergic antagonist did not directly enhance the behavioral expression of fear learning, as the fear responses collected during fear acquisition remained stable during memory reactivation on day 2, and did not affect US expectancy ratings. These findings demonstrate how noradrenergic stimulation critically influences fear generalization in humans. 

In a similar attempt, Lonsdorf et al. [132] hypothesized that the administration of the selective noradrenaline reuptake inhibitor reboxetine (RBX), which boosts the NA levels, could enhance extinction and prevent the reinstatement of fear. Participants (n = 42) were randomly assigned to the placebo group or the RBX group and underwent a fear-learning paradigm. The experimental design was carried out over three non-consecutive days: on day 1, the participants were fear-conditioned with two different CSs (i.e., discrete symbols) displayed intermittently in three different rooms used as context (CXT) and paired with mild electrotactile stimulation (US). On day 2, after the extinction learning phase, the participants received either a pill containing 4 mg of reboxetine or a placebo pill. On day 8, one week after fear acquisition, the participants performed a recall test in which the CSs were always presented without the US and a reinstatement phase after three unsignaled USs. The effects of fear acquisition and extinction were measured by means of SCR and functional magnetic resonance imaging (fMRI) in every test session. The results showed no group differences in SCR, but neuroimaging data from the recall test revealed greater activation in the subgenual part of the vmPFC and the posterior hippocampus in response to CS cues in the placebo group, in line with previous studies [22,37,44,101]. Instead, no increased activation in brain areas previously involved in the recall of extinction memory was observed in the RBX group. Moreover, after reinstatement, increased activation was observed within the left amygdala to the CS+ cue in the RBX group, whereas decreased activation in the vmPFC and the anterior hippocampus was seen in the placebo group. Thus, the authors suggested that extinction memory cannot be modulated by noradrenaline manipulations, as the stimulation of NAergic transmission prior to fear learning with reboxetine enhanced fear memories and ensured fear return (i.e., there were no effects on the consolidation of extinction memory).

In a subsequent study that investigated the effect of enhanced NA signals during fear extinction learning, Kausche et al. [133] administered α2-adrenergic receptor antagonist yohimbine to healthy volunteers. The participants (n = 125) were pseudo-randomly assigned to four experimental groups based on the type of medication administered. The authors decided to conduct a fear-learning paradigm on two consecutive days. On day 1, the participants underwent a fear-generalization paradigm. During the baseline session, eight neutral face stimuli were presented, and participants were asked to rate the intensity of an electric shock (US). The faces were shown on a circular structure. Thus, the two opposing faces, placed at 180°, were used as the CS+ and CS-. During the acquisition phase, both CSs were presented. Only one face was associated with the US (i.e., a mild electric shock), while the other face was never followed by the US. On day 2, based on group allocation, the participants received either a placebo, 20 mg of hydrocortisone, 20 mg of yohimbine (which enhanced noradrenergic stimulation), or both drugs before a test of fear generalization. Then, the complete set of stimuli was presented, but in random positions, and the participants were asked to indicate which face was paired with the US. SCR was used as an index of fear learning. The results showed that noradrenergic stimulation strengthened fear memory expression, as evidenced by greater responses to the CS+, while the responses to the CS- remained unaffected. These findings corroborated previous findings demonstrating how noradrenergic stimulation improves memory accuracy and contextual memory retrieval [125,126]. Moreover, as fear generalization is supposed to rely on the individual’s capacity to perceptually discriminate the CS+ from similar versions of the CS+ and CS-, these data showed that noradrenergic activation has the potential to influence this process, which is responsible for ensuring fear memory expression.

**Figure 2:**
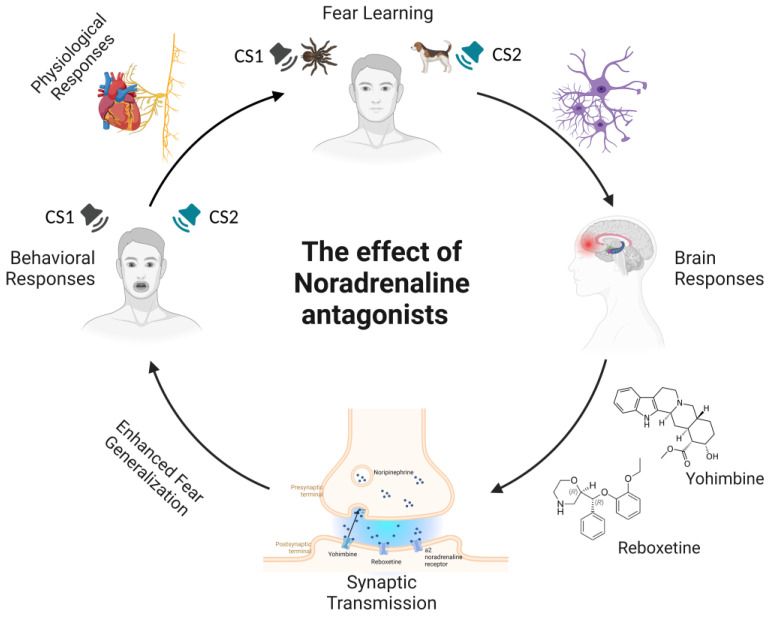
**Schematic representation of noradrenergic modulation during fear learning by the administration of adrenergic antagonists.** In fear-learning paradigms, following the pairing of a neutral stimulus with an innately aversive stimulus, conditioned fear responses develop after the presentation of the conditioned stimulus alone. Exposure to aversive events leads to the release of neurotransmitters, such as noradrenaline (NA). Specifically, NA is released from noradrenergic nerve terminals, where it diffuses across the synaptic cleft and activates adrenergic receptors to elicit a postsynaptic effect. Noradrenergic antagonists in presynaptic terminals act by decreasing the inhibitory influence of the α2-adrenoceptor on noradrenaline release. This causes the enhancement of fear memory and increased fear generalization, an active process that refers to an increased ability to discriminate between CS1 and CS2, leading to increased retrieval of fear memory and fear expression. Notes. CS1 = Conditioned stimulus 1; CS2 = Conditioned stimulus 2; NA = noradrenaline.

These findings revealed the role of noradrenergic modulation in extinction learning. Drugs stimulating adrenergic neurotransmission (i.e., reboxetine and yohimbine) actually delayed extinction learning by strengthening fear memory consolidation and triggering broader fear generalization (see Figure 2). Therefore, these studies indicate that noradrenaline modulation is an active process that may increase memory accuracy, affecting fear generalization processes (i.e., perceptual similarity between the CS+ and CS−) and thus increasing fear memory expression (see Table 2). 

## 4. The Effects of Fatty Acid Amide Hydrolase (FAAH) Inhibition

Increasing evidence suggests that pharmacological inhibition of the anandamide-degrading enzyme fatty acid amide hydrolase (FAAH) strengthens fear memory extinction and protects against the anxiogenic effects of stress [134,135]. Preliminary findings from animal studies have demonstrated that higher concentrations of the endogenous cannabinoid anandamide (AEA), caused by the inhibition of its main degradative enzyme FAAH, facilitates the extinction of fear memories [136,137,138]. In humans, genetic studies revealed that individuals with higher AEA showed enhanced extinction recall, and loss-of-function mutations at the human gene encoding FAAH have provided preliminary evidence that this reduced enzymatic activity may have beneficial effects on extinction learning and, consequently, the modulation of stress responses [139,140,141].

In a recent study, Mayo et al. [142] investigated how FAAH inhibition and higher baseline AEA influence fear learning in humans. In this study, participants (n = 45) were randomly assigned to the FAAH inhibitor group or the placebo group, and were tested on ten consecutive days. On day 1, participants were fear-conditioned with two colored lamps in two different contexts (i.e., two different rooms) used as CSs, and only one lamp was paired with an aversive auditory tone used as the US. Fear acquisition took place in one of the two contexts, and on the same day, extinction occurred 10 min after the acquisition phase in the context not used for acquisition. On day 2, the CS+ and the CS- were presented five times without the US in the same context being used during the extinction session, as a reminder of the fear memory trace, and then in the same context used for fear acquisition. Throughout the entire study, all participants received either 4 mg/day of a FAAH inhibitor (FAAHi) originally developed for analgesia, PF-04457845, or a placebo pill. In each session, fear responses were measured as changes in the eye-blink component of the startle response in facial EMG recorded from the zygomatic and corrugator muscles, as well as changes in physiological variables (i.e., SCR and heart rate). The results showed no effect of FAAH inhibition on the extinction of fear responses, but the FAAH group had a significantly lower startle response to the CS+ and SCR frequency on day 2, suggesting that FAAH inhibition enhanced the recall of extinction memory. Interestingly, these findings are compatible with rodent research, which has shown that the inhibition of FAAH does not affect within-session extinction, but enhances extinction memory consolidation, thus leading to increased fear suppression during extinction recall [143,144]. Moreover, participants who were treated with the FAAHi had reduced corrugator muscle reactivity, indicating that they were less stressed than the placebo group, which showed higher corrugator activation. 

Recently, another study [145] investigated the effects of pharmacological inhibition of the anandamide-degrading enzyme FAAH and the consequent accumulation of fatty acid amides. They administered the aryl piperazinyl urea inhibitor JNJ-42165279 to determine whether it altered neural activity in the amygdala during fear extinction. The authors adopted a 4-day experimental design that used fMRI to examine the effects of FAAH inhibition with the enzyme JNJ-42165279 on two behavioral tasks (i.e., an emotional face processing task and an inspiratory breathing load task) and a fear learning task. The participants (n = 43) were randomly assigned to the treatment group or the placebo group, and on day 4 of the experiment, before fMRI scanning, they underwent a fear-learning protocol. Firstly, the participants underwent a habituation phase, in which they were exposed five times to two fractal stimuli. Then, during fear acquisition, the two fractal stimuli were used as the CS+ and CS- and a loud noise was used as the US. Lastly, in the extinction phase, all the CSs were presented without the US. A single dose of 100 mg/day of JNJ-42165279 or a placebo was administered to participants, and their valence and arousal ratings of the CS cues on a five-point Likert scale after each experimental phase were used to assess fear responses. No differences between groups or between sessions were observed. Both groups were successfully fear-conditioned. Immediately after the acquisition phase, the valence and arousal ratings were higher for the CS+ than the CS-, while after extinction, the ratings were similar for both the CS+ and CS-. FAAH inhibition increased activation within the anterior cingulate and bilateral insula during fear acquisition, thus reflecting differences in neural activation between presentations of the CS+ and the CS-; however, it did not affect the within-session extinction learning, neither on a subjective level nor at the neural level. According to these findings, which contrast with the previous study by Mayo et al. [142], FAAH inhibition did not alter the extinction of fear responses. This is probably because the experimental paradigm used in this study was developed to enable the registration of the relevant neural circuitry, but did not allow the testing of fear consolidation and, therefore, how increases in AEA can affect the process of extinction learning. It still remains to be determined whether these effects will generalize to specific disease populations, most importantly to patients with PTSD, which further studies have been attempting to investigate [146,147].

**Figure 3:**
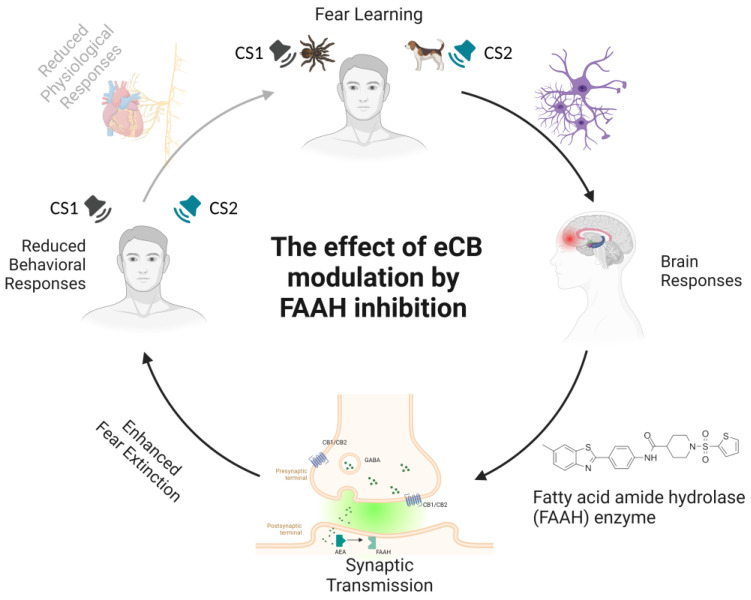
**Schematic representation of the effects of anandamide (AEA) elevation on fear extinction learning.** The acquisition of conditioned fear is achieved by presenting a stimulus paired with an aversive unconditioned event. As a result of this pairing, fear learning takes place, manifesting as the development of conditioned responses to the conditioned stimulus. The modulation of fear learning by the endocannabinoid (eCB) system occurs via cannabinoid receptor type 1 (CB1) signaling. The endocannabinoid transporter (ECT) mediates eCB synaptic re-uptake. The most important eCB, anandamide (AEA), acts as a low-efficacy agonist at CB1 receptors and is released after postsynaptic synthesis to retroactively bind to endocannabinoid receptors CB1 and CB2 at the presynaptic site. AEA is preferentially metabolized by fatty acid amide hydrolase (FAAH) into arachidonic acid and ethanolamine. Indeed, the inhibition of FAAH activity maintains AEA signaling during fear learning, favoring top-down cortical control of the amygdala and resulting in emotion regulation. Specific effects of the local inhibition of AEA in humans are reflected by an increased firing rate in prefrontal brain regions that facilitates prefrontal–amygdala connectivity through alterations in synaptic transmission. FAAH activity is expressed in enhanced fear extinction and selective attenuation of autonomic stress responses (i.e., reduced physiological and behavioral responses to fear stimuli). Notes. eCB = endocannabinoid system; FAAH = fatty acid amide hydrolase; CS1 = conditioned stimulus 1; CS2 = conditioned stimulus 2.

Although promising, these data yield contrasting results regarding the effects of AEA elevation via FAAH inhibition on the extinction of fear memories. In these studies, treatment with FAAH inhibitors did not affect the acquisition of fear, and even though Mayo et al. [142] showed the impact of reduced FAAH activity on extinction learning, that effect was not sufficiently robust (see Figure 3). Indeed, the implications of FAAH inhibition for stress-related behaviors remains an open question (see Table 3).

## 5. Discussion

Humans show strong sensitivity to potential threats [148,149,150,151,152], and predicting upcoming dangers is critical to an individual’s survival [3,153,154]. Indeed, from an evolutionary perspective, learned fear is vital to activate defensive behaviors in expectation of danger, increasing an organism’s likelihood of surviving [8,29,61]. In this context, extinction—in which the fear response to a conditioned stimulus decreases when the reinforcement is omitted—may be the simplest and most effective method of controlling emotional responses [101,132,155,156]. The regulation of emotional states and extinction of aversive memories have generated much interest in the past twenty years due to the important implications they may have in psychiatric settings. 

However, it is still questionable whether the mechanisms underlying fear extinction facilitation are based upon updating the original CS–US fear memory or inhibiting the original excitatory fear memory trace [10,101]. Understanding the potential of extinction-enhancing agents, which specifically alter regulatory systems, could, therefore, help us to understand how these agents (such as glutamate receptor agonists and endocannabinoid and noradrenaline antagonists) can enhance the extinction process and avoid the retention of aversive memories, which, in turn, have the potential to trigger trauma [157,158]. 

As discussed in previous sections, there is a large body of evidence suggesting that various neuropharmacological agents can interact with extinction learning to facilitate (or, in some cases, impair) the extinction of aversive memories. In particular, activating neurotransmitters and specific synaptic receptors is useful for promoting fear memory extinction, and highlights effects on fear- and stress-related behavior, physiological responses, and biochemistry in humans [143,144,159]. Crucially, after being activated, neurotransmitter receptors in synapses are dynamically modulated and actively redistributed, strengthening or weakening synaptic connections. Neurotransmitters and neuromodulators activate cellular kinase pathways that change synaptic strength, nerve conduction properties, and gene transcription profiles [160,161]. Other signaling pathways mobilize intracellular calcium ions and lipid mediators that have profound effects on neuronal functions, influencing the mechanisms underlying the extinction of fear memory [160,161]. Therefore, the suppression of conditioned fear responses following extinction training may result from a loss of synaptic modifications underlying the memory of the CS-US association formed during fear learning through different potential mechanisms involving brain plasticity [162,163,164,165,166,167,168]. A widely accepted hypothesis suggests that extinction depends on the formation of new associations competing with the original conditioned responses via plasticity at excitatory inputs to inhibitory interneurons or increased inhibition of principal cells in the bilateral amygdala [4]. Another possible explanation is a depotentiation of the thalamo-amygdala or cortico-amygdala synapses after fear learning [169]. 

Accordingly to Singewald et al. [170,171], pharmacological interventions targeting neurotransmitter systems, including serotonin, dopamine, noradrenaline, glutamate, and cannabinoids, as well as their downstream signaling pathways, can modulate synaptic plasticity, augment fear extinction, and strengthen extinction memory persistently in preclinical models [172]. Additionally, over the last decade, a plethora of newly identified molecular sites and receptors have been suggested to mediate the biological effects of metabolite changes in the hippocampus, amygdala, and posterior parietal cortex during extinction learning [173,174,175,176,177,178,179,180]. Thus, targeting specific neurobiological systems, such as the glutamatergic, noradrenergic, and eCB systems, is critical for identifying important neurochemical mediators in the extinction of aversive memories. Interestingly, we have described how agonists of NMDA glutamate receptors may enhance synaptic plasticity in such neural circuits by binding to glutaminergic sites, facilitating NMDA receptor activity and enhancing the neural processes involved in the extinction learning of conditioned fear [87,108]. Indeed, these excitatory neurotransmitters are specifically known to play a crucial role in synaptic plasticity associated with the long-term potentiation of synaptic transmission, and are involved in the neurobiological mechanisms of learning and memory, including hippocampus-dependent implicit learning and amygdala-dependent fear learning and fear extinction [181,182,183,184]. Furthermore, it has been shown how combined administration of DCS and VPA, used as adjunctive agents in cognitive–behavioral treatment for fear disorders, including anxiety disorders and PTSD, may facilitate the consolidation of fear memory extinction. Thus, when DCS and VPA are used as adjunctive agents, they simultaneously prevent the new acquisition of fear conditioning and the reinstatement of fear, decreasing the risk of relapse without enhancing anxiety or PTSD symptoms when similar aversive events are encountered [185,186]. 

Importantly, it emerged that enhancing noradrenergic signaling during extinction strengthened fear memory expression, indicating increased conditioned responses and a crucial generalization of fear expression. Increases in fear expression after noradrenergic stimulation could suggest that noradrenergic arousal may enhance fear memory expression and increase perceptual discrimination of CSs, therefore making noradrenaline necessary for the retrieval of contextual memory [128,187,188]. Moreover, noradrenergic arousal may increase the activity of areas implicated in enhanced responses to a conditioned stimulus, rather than areas involved in the extinction process, such as the amygdala or insular cortex [22,181,189,190]. Thus, it is possible that noradrenergic manipulation generally affects the consolidation of fear memory, as the key role of noradrenaline in increasing excitatory memory traces would be consistent with its role in the arousal-dependent enhancement of emotional memories [125]. From an evolutionary point of view, this would be an adaptive function enabling the preparation of appropriate future coping-related behavior. 

Furthermore, it is worth noting that the investigations into exogenous eCB system activation have demonstrated that CB receptors modulate fear-learning processes, particularly fear extinction [191,192]. Specifically, the pharmacological inhibition of FAAH enzyme activity prolongs the regulatory effects of the eCB system and reverses the stress-induced anxiety state in a cannabinoid receptor-dependent manner [192,193,194]. Available evidence shows that the inhibition of FAAH and the resulting accumulation of fatty acid amides may have anxiolytic effects in humans, which might be due to the accumulation of eCBs acting on the CB1 cannabinoid receptor [195,196]. Indeed, FAAH inhibition maintains higher endogenous cannabinoid anandamide (AEA) signaling during periods of stress, probably due to strengthened top-down cortical control of the amygdala, which attenuates emotional changes produced by stress and promotes the consolidation of fear extinction memory [197]. 

Finally, we suggest that the development of new therapeutics aimed at understanding the pathophysiology and potential treatment of anxiety disorders should be imperative: anxiety disorders are some of the most common psychiatric disorders, affecting more than 33% of the population during their lifetime [198] and, thus far, only psychotherapy and pharmacotherapy are typically used to treat them, often with disappointing outcomes [157,199]. This perspective, however, has been evolving in recent years. New and alternative methods for treating anxiety disorders have been developed, thanks to advances in our understanding of how pharmacological agents modulate extinction learning [170]. These new therapeutic approaches are built on the assumption that anxiety disorders, including phobias and post-traumatic stress disorder (PTSD), can be interpreted as the result of strong associative aversive learning, and clearly indicate that a broad range of drugs, acting through a wide variety of neurophysiological mechanisms, can alter such learning, sometimes in a lasting manner [200,201,202]. So far, it is acknowledged that a significant percentage of anxiety patients do not respond successfully to the current treatments, including anxiolytic pharmacotherapy and cognitive–behavioral therapy [33,132], and show a high probability of chronicity or experience a return of fear. Therefore, one common clinical method is to combine psychotherapy with adjuvant pharmacological therapies. These enhancers have been experimentally and clinically tested, with promising results; most studies confirmed that using NMDA agonists and cannabinoids as pharmacotherapies increased the efficacy of extinction-based psychotherapies [33]. Furthermore, new cutting-edge avenues for clinical research would be to combine these treatments with non-invasive brain stimulation techniques (NIBS) that could target specific brain areas involved in fear acquisition and modulate the functional mechanisms behind aberrant fear learning (i.e., PTSD or anxiety) [6,13,29,61]. In particular, NIBS interventions with transcranial magnetic stimulation (TMS) and transcranial direct current stimulation (tDCS) have achieved optimal results in targeting brain nodes to selectively interfere with fear learning [13,26,29,203,204,205,206]. Currently, such techniques have been used to modulate cerebral activity during the consolidation and extinction of fear memories, with the ultimate goal of modulating these processes, which are aberrant in different pathological fear states caused by trauma, stress, and anxiety [6]. Thus, given this promising evidence of the use of neuropharmacological adjuvants combined with the use of brain neurostimulation techniques, it is possible to hypothesize better results regarding the speed of recovery from a psychiatric disorder, as well as the long-term effect of these manipulations. 

In conclusion, gaining a further understanding of how neuropharmacological agents act on major neurotransmitter systems to promote long-term potentiation of fear extinction may provide relatively safe and potentially effective means for treating individuals with trauma-related diseases. This possibility further underlines the importance of using pharmacological enhancers to optimize therapies in those patients for whom classic psychotherapy approaches alone fail to produce significant psychological improvements. However, there is a crucial question still to be answered: what about the specificity of these neuropharmacological agents for different brain functions? How do these enhancers exert specific effects only on fear memories, on emotional memories in general, on other types of memory, and/or on other related cognitive processes (i.e., attention, working memory, and executive functions)? It is still difficult to answer these questions, so the protocols described in this review, although using different conditions and manipulations, do not directly investigate the involvement of other cognitive processes. Moving forward, it will be important to investigate, in a combined way, the effects that the agents specifically have on aversive memories related to cognitive functions. Additionally, as the studies included in this review focused only on pharmacological methods to facilitate fear extinction learning in healthy participants, it would be interesting to assess the effects and mechanisms of the action of pharmacological adjuvants that foster the extinction of fear memories in clinical populations, such as psychiatric or brain-damaged patients.

Finally, solid work will be needed to gain a deeper circuit-level understanding of how enhancing specific neurobiological systems acts on distinct components of the neuronal circuitry underlying memory extinction. This would provide a first step toward the regulation of maladaptive fear memories and improvement of extinction-dependent learning. 

## Figures and Tables

**Table 1:** Summary of findings in studies with the administration of NMDA agonist DCS.

Study	Group (N)	Pharmacological Treatment	Mechanism of Action	Experimental Paradigm	Phase of Fear Learning	CSs	US	Psychophysiological Measure	Main Findings
Kuriyama et al. [111]	DCS treatment (16) VPA treatment (14) Combined VPA–DCS treatment (15) Placebo treatment (15)	100 mg of powdered DCS 400 mg of granulated VPA 1200 mg of lactose (placebo)	DCS is an agonist of the NMDA receptor VPA is an inhibitor of GABA transaminase and HDAC	3 days	Acquisition Extinction Recall Reinstatement	Geometric figures	Electric shock	SCR	DCS and VPA enhanced extinction learning of the fear CRs in the post-recall reinstatement phase, but not in the extinction recall phase
Kuriyama et al. [116]	DCS × Sleep treatment (14) DCS × Wake treatment (14) VPA × Sleep treatment (15) VPA × Wake treatment (15) Placebo × Sleep treatment (15) Placebo × Wake treatment (14)	100 mg of powdered DCS 400 mg of granulated VPA 1000 mg of lactose (placebo)	DCS is an agonist of the NMDA receptor VPA is an inhibitor of GABA transaminase and HDAC	1 day	Acquisition Extinction Recall Reinstatement	Geometric figures	Electric shock	SCR	VPA treatment reduced fear CRs in the extinction and acquisition phases during the second learning session DCS treatment blocked the effect of the reinforced CS–US pairing only in the waking group; VPA blocked the effect of the reinforced CS–US pairing only in the sleep group
Ebrahimi et al. [116]	DCS treatment group (17) Placebo treatment (20)	50 mg of powdered DCS Placebo	DCS is an agonist of the NMDA receptor	3 days	Habituation Acquisition Extinction Recall	Ekman faces	Auditory tone	SCR and fMRI	DCS administration enhanced extinction memory retention by preventing differential CRs from extinction learning to recall in subjective arousal ratings and attenuating BOLD responses in the hippocampus and amygdala

Notes. DCS = D-cycloserine; VPA = valproic acid; GABA = gamma-aminobutyric acid; HDAC = histone deacetylases; CS–US = conditioned stimulus–unconditioned stimulus; CRs = conditioned responses; NMDA = N-methyl-D-aspartate; SCR = skin conductance response; fMRI = functional magnetic resonance imaging.

**Table 2:** Summary of findings in studies with the administration of noradrenaline antagonists.

Study	Group (N)	Pharmacological Treatment	Mechanism of Action	Experimental Paradigm	Phase of Fear Learning	CSs	US	Psychophysiological Measure	Main Findings
Lonsdorf et al. [133]	RBX treatment (23) Placebo treatment (19)	4 mg of RBX Placebo	Reboxetine is an inhibitor of noradrenaline reuptake	3 days	Acquisition Extinction	Symbols shown in three different contexts	Electrotactile shock	SCR and fMRI	No SCR differences between groups at the behavioral level Before reinstatement, only the placebo group showed higher activation in vmPFC for CS cues After reinstatement, the RBX group showed higher amygdala activation for CS cues
Kausche et al. [134]	Placebo treatment (31) CORT treatment (31) YOH treatment (34) CORT+YOH treatment (29)	20 mg of cortisol 20 mg of YOH 20 mg of cortisol and YOH Placebo	Cortisol is an agonist of glucocorticoid receptor and annexin A1 Yohimbine is an α2-adrenergic blocking agent	2 days	Habituation Acquisition	Eight neutral faces	Electric shock	SCR	The YOH group showed higher SCR across a similarity continuum from CS+ to CS− (increased responses to the CS+) Cortisol did not enhance fear memory expression, but increased fear generalization
Soeter and Kindt [132]	Yohimbine treatment (20) Propranolol treatment (20)	40 mg of YOH 20 mg of propranolol Placebo	Yohimbine is an α2-adrenergic blocking agent	3 days	Acquisition Recall Extinction	Three images	Auditory tone	SCR Systolic and diastolic blood pressure Amylase level	The YOH group showed higher startle fear responses during fear memory reactivation on day 2 The propanolol group showed a reduction in startle fear responses during reconsolidation and extinction

Notes. RBX = reboxetine; CORT = cortisol; YOH = yohimbine; CS+ = conditioned stimulus; CS− = control stimulus; SCR = skin conductance response; fMRI = functional magnetic resonance imaging; vmPFC = ventromedial prefrontal cortex.

**Table 3:** Summary of findings in studies with AEA elevation by FAAH inhibition.

Study	Group (N)	Pharmacological Treatment	Mechanism of Action	Experimental Paradigm	Phase of fear Learning	CSs	US	Psychophysiological Measure	Main Findings
Mayo et al. [143]	FAAH inhibitor treatment (16) Placebo treatment (29)	4 mg/day of PF-04457845 (FAAH inhibitor) Placebo	PF-04457845 is an inhibitor of FAAH	10 days	Habituation Acquisition Extinction Recall Renewal	Two lamps shown in two different contexts	Auditory tone	SCR, ECG, and EMG	The FAAH inhibitor group showed lower responses to the CS+ on day 2, indicating enhanced recall of extinction memory
Paulus et al. [146]	FAAH inhibitor treatment (22) Placebo treatment (21)	100 mg/day of JNJ-42165279 (FAAH inhibitor) Placebo	JNJ-42165279 is an inhibitor of FAAH	4 days	Habituation Acquisition Extinction	Fractal stimuli	Auditory tone	fMRI	No differences between groups during the acquisition and extinction phases

Notes. FAAH = fatty acid amide hydrolase; SCR = skin conductance response; ECG = electrocardiogram; EMG = electromyography; fMRI = functional magnetic resonance imaging.

## Data Availability

Not applicable.

## Abbreviations

NS	neutral stimulus
US	unconditioned stimulus
CS+	conditioned stimulus
CS-	conditioned stimulus never paired with the US
CXT	conditioned context
CR	conditioned response
SCR	skin conductance response
EMG	electromyography
fMRI	functional magnetic resonance imaging
BOLD	blood-oxygen-level-dependent
PTSD	post-traumatic stress disorder
NMDA	N-methyl-D-aspartate
AMPA	α-amino-3-hydroxy-5-methyl-4-isoxazole propionate
DCS	D-cycloserine
VPA	valproic acid
NA	noradrenaline
YOH	yohimbine
RBX	reboxetine
eCBs	endocannabinoids
AEA	anandamide
FAAH	fatty acid amide hydrolase
FAAHi	FAAH inhibitor
MAGL	monoacylglycerol lipase
